# Standardization of 16S rRNA gene sequencing using nanopore long read sequencing technology for clinical diagnosis of culture negative infections

**DOI:** 10.3389/fcimb.2025.1517208

**Published:** 2025-03-06

**Authors:** Ian Butler, Olivia Turner, Kulsoom Mohammed, Mazeda Akhtar, Daniel Evans, Jonathan Lambourne, Kathryn Harris, Denise M. O'Sullivan, Chrysi Sergaki

**Affiliations:** 1 Department of Microbiology, National Health Services (NHS) East and South East London Pathology Partnership, Bart’s Health NHS Trust, London, United Kingdom; 2 Faculty of Biology, Medicine and Health, The University of Manchester, Manchester, United Kingdom; 3 Molecular and Cell Biology, National Measurement Laboratory (NML), Laboratory of the Government Chemist (LGC), London, United Kingdom; 4 School of Biosciences & Medicine, University of Surrey, Guildford, United Kingdom; 5 Medicines and Healthcare Products Regulatory Agency (MHRA), London, United Kingdom

**Keywords:** 16S rRNA, Oxford nanopore, long read sequencing, standardization, culture negative

## Abstract

The integration of long-read sequencing technology, such as nanopore sequencing technology [Oxford Nanopore Technologies (ONT)], into routine diagnostic laboratories has the potential to transform bacterial infection diagnostics and improve patient management. Analysis of amplicons from long-read sequencing of the 16S rRNA gene generates a comprehensive view of the microbial community within clinical samples, significantly enhancing sensitivity and capacity to analyse mixed bacterial populations compared to short read sequencing approaches. This study evaluates various ONT sequencing approaches and library preparation kits to establish a reliable testing and quality framework for clinical implementation. This study highlights the critical importance of using well-characterized reference materials in validating and revalidating long-read sequencing methods, leveraging a combination of standardized reference materials and clinical samples to navigate the evolving landscape of microbial diagnostics. It presents a robust validation framework for laboratory accreditation and outlines a methodology for comparing the performance of newer ONT chemistries with earlier versions. Additionally, the study details the methods and quality control measures necessary for achieving more accurate and efficient diagnoses of bacterial infections, ultimately reducing time to treatment and enhancing patient outcomes.

## Introduction

Amplicon-based sequencing techniques targeting the 16S ribosomal RNA gene (16S rRNA) are crucial for identifying bacterial pathogens, particularly in culture-negative samples (Harris and Brown, 2022; Hartley and Harris, 2014). This approach enhances patient care by enabling definitive diagnoses and guiding patient management. Clinically, 16S rRNA gene sequencing is invaluable for detecting bacteria that failed to grow from clinical samples typically due to prior empirical antibiotic treatment, particularly in critical infections such as meningitis, pneumonia, osteo-articular infection and sepsis (Martínez et al., 2020; Mcgill et al., 2016). This method also enables the detection of fastidious or slow-growing organisms that are challenging to cultivate using standard methods (De Melo Oliveira et al., 2013). Early identification of the causative agent reduces morbidity and mortality, while delays in diagnosis are associated with increased healthcare costs from broad-spectrum antibiotic use, inpatient admissions, and nursing resources (Hollenbeak et al., 2023).

16S rRNA gene PCR and subsequent sequencing of the amplicon is performed on culture-negative samples from anatomically sterile sites (e.g. tissue biopsies, cerebrospinal fluid), in a small number of NHS (National Health Service) laboratories in the UK using unstandardized protocols (Harris and Brown, 2022; Lampejo et al., 2021). Variations in sample processing, extraction methods, primer design, and instrumentation can result in significant inter-laboratory discrepancies in assay performance and accuracy. Moreover, the lack of commercially available internal and external quality assurance materials complicates efforts to maintain and monitor quality. Consequently, laboratories may find it challenging to introduce and validate in-house 16S rRNA gene sequencing services, choosing instead the easier but more costly approach of sending samples to referral laboratories. This approach incurs inflated costs and turnaround times of often more than one week, which in turn delays diagnosis and appropriate patient management. Short-read sequencing technologies remain the gold standard for sequencing in both research and clinical laboratories. Although Illumina short-read sequencing is predominantly used in research, its adoption in clinical diagnostic laboratories is limited due to high costs and throughput requirements. Instead, Sanger sequencing serves as the primary method for 16S rRNA gene analysis in diagnostic laboratories in the UK. Sanger sequencing has significant limitations in flexibility, sensitivity, and performance, particularly for sequencing the full 16S rRNA gene or resolving polymicrobial infections (Kumar et al., 2019). This reliance underscores the need for alternative methods and the establishment of robust testing and quality frameworks for newer more cost-effective sequencing technologies to ensure consistent, high-quality results across healthcare settings.

Long-read sequencing from Oxford Nanopore Technology (ONT) provides enhanced speed, scalability, and sensitivity compared to traditional short-read methods (Petersen et al., 2019). While alternative long-read sequencing options like PacBio are available, their prohibitive costs make them impractical for clinical laboratory settings, severely restricting their use. In contrast, the compact size, low cost, and flexible throughput requirements of ONT make it an appealing choice for routine clinical microbiology laboratories. By enabling amplicon-based metagenomic analysis of microbial communities, ONT enhances the characterization of polymicrobial infections—an area where traditional short read sequencing methods struggles due to the difficulty in interpreting mixed electropherogram patterns. Consequently, the number of PCR cycles in conventional 16S rRNA gene short-read sequencing protocols must be carefully limited to prevent non-specific over-amplification of low-abundance environmental microorganisms. Such over-amplification can result in mixed sequencing signals and produce ambiguous or unreadable electropherograms, ultimately reducing diagnostic sensitivity. Moreover, ONT can sequence the entire ~1500 bp of the 16S rRNA gene, addressing the limitations of short-read technologies. An in-house long-read sequencing service for 16S rRNA gene analyses will improve bacterial identification in clinical samples, enabling definitive diagnoses and facilitating the transition to narrow-spectrum antimicrobials thus supporting antimicrobial stewardship.

This study aims to develop a comprehensive end-to-end testing methodology and a robust quality framework for 16S rRNA gene amplicon sequencing using Oxford Nanopore Technologies (ONT), with the ultimate goal of acheiving ISO:15189 accreditation under the United Kingdom Accreditation Service (UKAS). To ensure method standardization, characterized DNA and whole-cell control materials obtained from two of the UK National Laboratories; National Measurement Laboratory (NML) and the Medicines and Health Regulatory Agency (MHRA), were evaluated, alongside the various sequencing approaches (O'sullivan et al., 2021; Sergaki et al., 2022; Anwar et al., 2023; Amos et al., 2020). This pioneering study is the first to employ characterized reference materials alongside clinical samples to rigorously validate various ONT chemistries and sequencing approaches for clinical diagnostics. This study seeks to equip laboratories with the necessary tools and confidence to develop and implement a standardized long read 16S rRNA gene sequencing service, ensuring consistency and reliability in diagnostic applications.

## Materials and methods

### Sample material

#### NML metagenomic control materials MCM2α and MCM2β

The metagenomic control materials (MCM) 2α and β, containing genomic DNA from mixtures of different microbes, were previously developed by the UK National Measurement Laboratory (NML) (O'sullivan et al., 2021). These materials were used in this study to assess PCR and sequencing efficiency and accuracy. MCM2α and MCM2β contain DNA extracted from fourteen clinically relevant bacterial organisms in variable concentrations, expressed as copies per µL, as previously determined by NML. For microbial composition of both MCM2 materials (**Supplementary Data 1**).

#### World Health Organization international reference reagents for microbiome

The World Health Organization (WHO) international whole cell reference reagent for DNA extraction of the gut microbiome, WC-Gut RR, (NIBSC 22/210) was obtained from the UK Medicines and Health and Regulatory Agency (MHRA) and used in this study to assess extraction efficiency and bias (Anwar et al., 2023; Sergaki et al., 2022). Twenty bacterial species are present in equal abundance in the whole cell material. The WHO DNA-Gut reference reagent (NIBSC 20/302) which has the same microbial composition as the whole cell standard was also used in this study to assess accuracy of bioinformatic analysis pipelines (Amos et al., 2020). For microbial composition of WHO WC-Gut RR material (**Supplementary Data 2**).

#### Clinical samples

DNA extracts from clinical samples were obtained from the microbiology laboratory of The Royal London Hospital from patients who have had previous 16S rRNA gene sequencing requests from culture-negative samples from ‘sterile’ anatomical sites; tissue, pus, cerebrospinal fluid (CSF), joint fluid and pleural fluid. Ethical approval was not sought as samples were taken as part of routine clinical care, and the samples used in this study were those that were surplus to clinical requirement, once routine diagnostic work-up had been completed. Clinical samples were anonymized and used in this study to assess the sensitivity and specificity of ONT sequencing versus Sanger sequencing and to compare reproducibility of clinical results. Additional factors, such as ease of use, laboratory workflow, result turnaround times, and improved sequencing capabilities in identifying mono and polymicrobial infections were also considered.

### Sample processing

#### Metagenomic control material (MCM2α and MCM2β)

Dilutions of MCM2α and MCM2β material were prepared in triplicate at the Royal London Hospital Microbiology Department as follows:

A total of eight vials of MCM2α material, each containing 25μL were pooled to a total volume of 200μl, followed by vortexing and pulse centrifugation. A volume of 50μL of MCM2α was then aliquoted to four separate vials. A 1:10 dilution was prepared by adding 20μL of MCM2α to 180μL of nuclease free water followed by vortexing and pulse centrifugation. A volume of 50μL of MCM2α 1:10 were aliquoted to four separate vials. A 1:100 dilution was prepared by adding 20μL of MCM2α 1:10 to 180μL of nuclease free water followed by vortexing and pulse centrifugation. A volume of 50μL of MCM2α 1:100 was aliquoted to four separate vials. The same procedure was repeated for MCM2β, and all vials were stored at -20°C.

#### WHO international reference reagents for microbiome

Lyophilized WHO reference reagents were reconstituted in 600µL of Phosphate Buffered Saline (PBS) (ThermoFisher Scientific, 10010023) as per manufacturer’s instructions.

#### Clinical samples

As per local standard operating procedures, all clinical samples were subjected to bead beating using Lysing Matrix E tubes (MP Bio, 6914100) and QIAGEN TissueLyser set at 50 oscillations per second for 2 minutes. Tissue samples were pre-processed by emulsifying with Tissue Lysis Buffer ATL in a 1:1 ratio (Qiagen, 939011) and 20µL of proteinase K (Qiagen, RP107B-1) for 2 hours at 56°C before bead-beating. DNA was extracted using the AusDiagnostics MT-Prep as described in the next section. All samples previously underwent 16S rRNA gene PCR and Sanger sequencing as part of routine testing (Lampejo et al., 2021).

### DNA extraction

#### DNA extraction methods

The performances of four DNA extraction methodologies were validated in this study. A volume of 200µL of sample material was extracted and eluted into a final volume of 50-60µL for each extraction method. The four extraction kits were as follows: QIAmp DNA/Blood kit (Qiagen, 51104), EZ1&2 Virus Mini kit v2.0 (Qiagen, 955134), EZ1&2 DNA Tissue kit (Qiagen, 953034), and MT-Prep Nucleic Acid Extraction kit (AusDiagnostics, 93010). All extraction methods were conducted as per manufacturer’s instructions.

EZ2 Virus Mini kit and EZ2 DNA Tissue kits were used on the EZ2 Connect extraction platform and eluted into 60µL (Qiagen, 9003210), the MT-Prep Virus/Pathogen kit was used on the Aus Diagnostics MT-Prep 24 extraction platform and eluted into 50µL (AusDiagnostics, 7570a8549b11). These three extraction methods employ magnetic bead extraction of nucleic acid from sample material. Manual extraction of nucleic acid extraction using a spin column method was performed using the Qiagen QIAmp DNA/Blood kit and eluted into 50µL.

#### Quantification and quality assessment of nucleic acid

WHO WC-Gut RR was extracted in triplicate by all four extraction methods. All DNA extracts underwent a 1:1.8 bead clean up with AMPure XP beads for purification (Beckman Coulter, A63880) and DNA quantified using Qubit fluorimeter and Qubit dsDNA High Sensitivity kit (Invitrogen, 10616763). DNA purity was measured using the NanoDrop Microvolume Spectrophotometer (ThermoFisher Scientific), with an acceptable 260/280 ratio ≥1.7. DNA extracts were normalized before amplification to a concentration of 10ng in 15µL of nuclease free water. Normalized extracts were amplified and barcoded using the ONT 16S Barcoding all-in-one kit (Oxford Nanopore, SQK-16S024) as per manufacturer's instructions (maximum cycle number of 25 cycles).

#### Investigation of nucleic acid by digital PCR

To assess the yield of target amplicon, the WHO WC-Gut RR DNA extracts were sent to NML for quantification by digital PCR (copies/mL), targeting both the 16S rRNA gene and the *E. coli* target gene (*uid*A). Digital PCR was performed using QX200 digital PCR instrument (Bio-Rad, 1854001). Further information is provided in the **Supplementary Data 3**.

### PCR, library preparation and sequencing

Library preparation for reference materials and clinical samples was performed using the ONT 16S Barcoding all-in-one kit (Oxford Nanopore, SQK-16S024/SQK-16S114.24). The ONT 16S Barcoding all-in-one kit only employs primers targeting the V1-V9 region (primer sequences not disclosed by manufacturer) and has a PCR (Polymerase Chain Reaction) cycle number of 25 cycles. Library preparation was performed as per manufacturer’s instructions but with two amendments. An additional 1:1 library clean-up with AMPure XP beads (Beckman Coulter, A63880) was included to concentrate the final library. In addition, nuclease free water (Thermo Scientific, R05810) was used as the elution buffer instead of Tris-HCl pH 8.0 with 50 mM NaCl and the library was eluted into a final volume of 17μL with a recommended concentration of 50-100 fmoles.

ONT MinION flow cells (Oxford Nanopore, FLO-MIN106D, R9 chemistry) and Flongle flow cells (Oxford Nanopore, FLO.FLG001.6, R9 chemistry) were used in the initial stages of this study. The updated Q20+ chemistry (Kit v14) was used when this became available; ONT 16S Barcoding all-in-one kit (v14) chemistry (Oxford Nanopore, SQK-16S114.14) and MinION flow cell and Flongle flow cells (Oxford Nanopore, FLO-MIN114, R10.4.1 chemistry). All MinION flow cells and Flongle flow cells were primed and loaded as per the manufacturer’s instructions. The reference materials used in this study were analyzed using both ONT kit chemistries (R9 and R10.4.1) across MinION flow cell and Flongle flow cell sequencing approaches for comparative purposes. To ensure the integrity of the results, reference materials and clinical samples were not combined within the same sequencing runs. Testing was conducted sequentially, with MCM2 materials analyzed first for comparative assessments, followed by the WHO-Gut-RR materials, and finally clinical samples. Each sequencing run was performed in triplicate at different time points to account for potential stochastic variability.

An additional in-house sequencing protocol termed the ‘16S ONT RBK method’ was developed and used to test reference materials and clinical samples. The 16S ONT RBK method involved performing two separate PCR reactions to amplify the V1-V2 and V1-V9 regions, respectively, of the 16S rRNA gene using in-house laboratory developed primers with a PCR cycle number of 40 cycles (Harris and Hartley, 2003; Dos Santos et al., 2019). In-house primers were obtained ‘LabReady’, normalized to 100uM in IDTE pH 8.0 [IDTE Buffer pH 8.0 (10 mM Tris-HCl/0.1 mM EDTA)] (Integrated DNA Technologies).

#### In-house primers targeting 16S variable regions: V1 - V2 - ~320bp

16S-22F: (Forward A): 5’- GCT CAG ATT GAA CGC TGG-3'

16S-22F: (Forward B): 5’- GCT CAG GAY GAA CGC TGG-3'

16S-358R: (Reverse) 5’- TAC TGC TGC CTC CCG TA-3'

The PCR cycling conditions for 16S V1–V2 PCR; initial denaturation at 94°C for 30 seconds, followed by 40 cycles of: denaturation at 94°C for 30 seconds, annealing at 55°C for 40 seconds and extension at 72°C for 1 minute with a final extension at 72°C for 10 minutes.

#### In-house primers targeting 16S variable regions: V1 - V9 - ~1450bp

16S-27F: (Forward): 5'-AGAGTTTGATCMTGGCTCAG-3'

16S-1492R: (Reverse): 5'-TACGGYTACCTTGTTACGACTT-3'

The PCR cycling conditions for 16S V1–V9 PCR; initial denaturation at 94°C for 30 seconds, followed by 40 cycles of: denaturation at 95°C for 30 seconds, annealing at 55°C for 40 seconds and extension at 65°C for 1.5 minutes with a final extension at 65°C for 10 minutes.

Amplicons from both PCR reactions were pooled before library preparation with the ONT Rapid Barcoding kit (SQK-RBK114.24). All PCR amplicons from both library preparation methods were analysed using the Tapestation D5000 screen tape (Agilent, 5067-5588) on the Tapestation System, 4150 (Agilent, G2992AA). To assess cross contamination during library preparation, three non-template controls (nuclease free water) were included in all sequencing experiments in positions 1, 15 and 24.

### Bioinformatic analysis

BugSeq bioinformatic analysis pipeline was used throughout this study. BugSeq is an online cloud based bioinformatic platform where FastQ files can be uploaded with results generated within clinically actionable timeframes. BugSeq 16S analysis (v5.0) uses NanoCLUST, a tool that performs a read clustering step, based on Uniform Manifold Approximation and Projection (UMAP), to generate a polished read which is then identified using QIIME2’s VSEARCH-based for greater accuracy. To minimise the occurrence of chimeras, BugSeq (v5.0) requires both 80% query coverage and 80% sequence identity to classify consensus sequences and employs a minimal cluster size threshold of 30 reads. Additionally, during pre-processing, BugSeq 16S filters out reads shorter than 1000 bp and longer than 1850 bp (Jung and Chorlton, 2021; Rodríguez-Pérez et al., 2021).

The Oxford Nanopore EPI2ME Desktop Agent (version 3.7.3) and the ‘FastQ 16S’ pipeline were employed in this study for the taxonomic identification of bacteria from clinical samples. The EPI2ME Desktop Agent ‘FastQ 16S’ analysis pipeline utilizes the NCBI Reference Sequence (RefSeq) database for sequence classification. RefSeq is an open access, curated, and annotated collection of publicly available nucleotide sequences. EPI2ME employs the BLAST algorithm for taxonomic assignment, quality control, and demultiplexing.

The EPI2ME Desktop Agent was selected for the analysis of clinical samples due to the incompatibility of BugSeq (v5.0) with the shorter read lengths generated by the in-house ONT Rapid Barcoding Kit (RBK) method. At the time of writing, BugSeq (v5.0) required the full 1500 bp 16S rRNA amplicon for accurate taxonomic identification. The ONT Rapid Barcoding Kit employs a transposase to simultaneously fragment template molecules and attach barcoded tags to the cleaved ends, resulting in shorter read lengths that were incompatible with BugSeq (v5.0). Since completing this study, an updated version, BugSeq (v5.4), has been released, which now supports shorter read lengths. This advancement will be considered in future studies to enhance taxonomic identification workflows

Towards the end of this study, the ONT EPI2ME Desktop Agent was decommissioned, and a new EPI2ME platform, developed by Metrichor Ltd. (a subsidiary of ONT), was introduced. The EPI2ME platform provides a variety of workflows for comprehensive nanopore data analysis. Specifically, the EPI2ME ‘wf-16S’ workflow was employed in the final stages of this study to classify 16S amplicons from WHO Gut RR material. This was done using the minimap2 approach alongside the built-in NCBI 16S/18S database.

To confirm species identification, the ONT EPI2ME platform ‘Alignment’ workflow was used to align raw reads against 16S reference sequences obtained from the NCBI Reference Sequence Database. The quality of these alignments was evaluated based on the depth and coverage of the 16S reference gene. A secondary quality check was performed by analyzing the bamstats CSV file produced during the minimap2 alignment to assess the length of mapped reads and their percentage coverage of the gene (**Supplementary Data 4A–D**).

#### Basecalling and sequencing quality

For the clinical sample study, the ONT MinKNOW software was utilized for data acquisition, basecalling, real-time analysis, and monitoring sequencing performance and quality. Key quality parameters included an acceptable Quality Score of >10 and a balanced ratio of passed reads to total reads. Additional quality control metrics, such as consistent pore saturation, translocation speed, and temperature, were monitored to maintain optimal sequencing conditions. The minimum read threshold was set at 200bp, at a run time of 12 hours (as per manufacturer’s instruction), and base called with super-accurate basecalling. Manufacturers claimed basecalling accuracy ranges from 97–97.5% for R9.4.1 chemistry and > 99% for R10.4.1 chemistry.

### Statistical analysis

To compare the relative abundance (RA) to actual abundance (AA) aka the ‘ground truth’ of bacterial organisms in the WHO WC-Gut RR material (**Supplementary Data 2**), four key reporting measures were calculated using the averages for each bacterial organism in the reference reagents without the inclusion of any contaminants found present in the samples as previously described (Anwar et al., 2023; Amos et al., 2020; Sergaki et al., 2022). The four key reporting measures include sensitivity (how many species are correctly identified), false positive relative abundance (FPRA) (relative abundance of false positives), diversity (total number of species detected) and similarity (Bray-Curtis Dissimilarity statistical measure used to assess the relative abundance of organisms in the comparison to the expected actual abundance). The equations for the four key reporting measures are below:


$$\text{Sensitivity}=\frac{\text{Number of Correctly Identified Species }}{\text{Total Number of Species in Reagent}}\times \;100$$

$$\text{False Positive Relative Abundance}=\frac{\text{Abundance of All False Positive Species}}{\text{Total Abundance of All Species}}\;x\;100$$

Diversity=Total Number of All Oserved Species (True Positive+False Positive)

$$\text{Similarity}\;\left[jk\right]=1-\;\frac{\text{sumabs }\left(\text{x}\left[\text{ij}\right]-\text{x}\left[\text{ik}\right]\right)}{\text{sum}\left(\text{x}\left[\text{ij}\right]+\text{x}\left[\text{ik}\right]\right))}$$

Where x[ij] and x[ik] refer to the quantity on species [i] in the actual species composition [j] and observed species composition [k] of the reagent. The vegdist function of the R vegan package (method = ‘bray’) can be used to calculate Bray-Curtis dissimilarity and was used in the current study, with 1-dissimlarity being used to calculate similarity.

The Kruskal-Wallis test was used to assess statistically significant changes in the RA of organisms in the MCM2α/β between runs.

Statistical analysis was performed using one-way ANOVA to compare the DNA yields across four extraction methods. *Post-hoc* pairwise comparisons were conducted using Tukey's Honestly Significant Difference (HSD) test to identify specific differences between methods. A significance level of 0.05 was used for all tests.

## Results

### Comparison of abundance in metagenomic control material

#### Identifying bias and limit of detection

**Figure 1:**
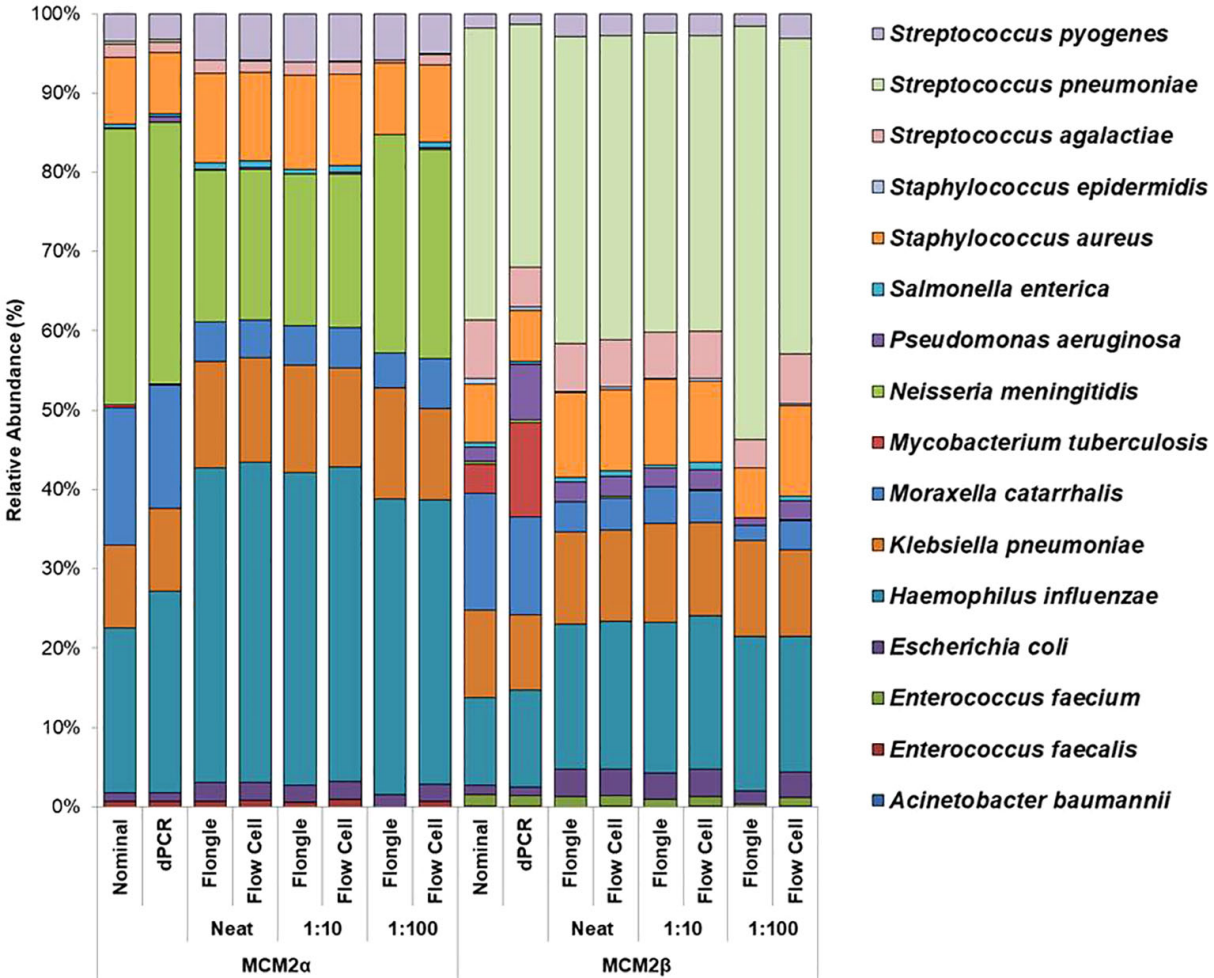
Average relative abundance (%) of bacterial organisms present in MCM2α and MCM2β obtained when by ONT sequencing using either the R9 MinION flow cell or the R9 Flongle flow cell. Three concentrations of the MCM2α and MCM2β materials were tested in triplicate (neat, 1:10 and 1:100) and run in parallel on each of the ONT sequencing approaches. The species abundance (%) of each dilution is reported, including the nominal and dPCR reported composition for the two materials (**Supplementary Data 3**).

**Figure 2:**
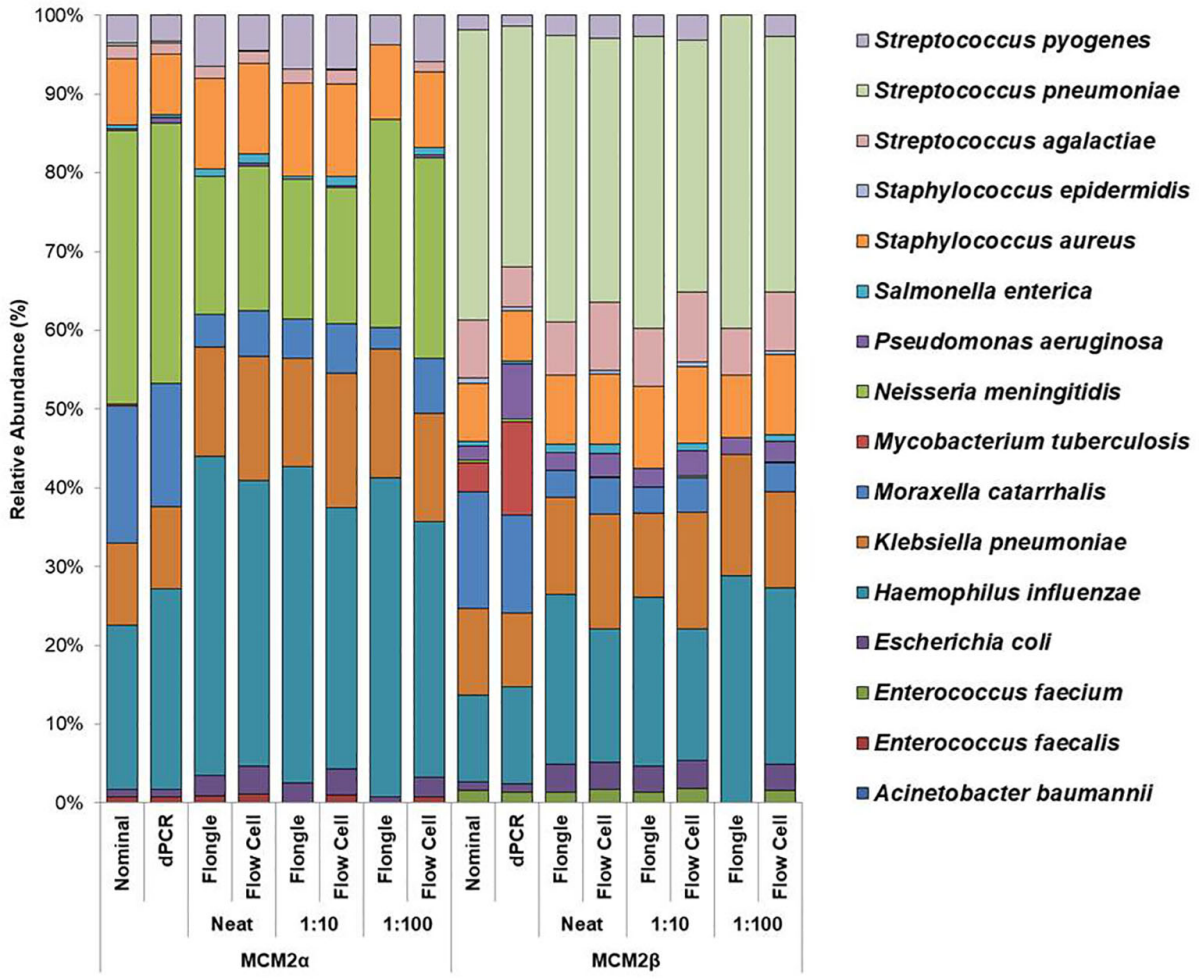
Average relative abundance (%) of bacterial organisms present in MCM2α and MCM2β obtained by ONT sequencing using either the R10.4.1 MinION flow cell or the R10 Flongle flow cell and version 14 kit chemistry. Three concentrations of the MCM2α and MCM2β materials were tested in triplicate (neat, 1:10 and 1:100) and run in parallel on each of the ONT sequencing approaches. The species abundance (%) of each dilution is reported, including the nominal and dPCR reported composition for the two materials (**Supplementary Data 3**).

Mock community materials, MCM2α and MCM2β, containing fourteen clinically significant bacterial organisms in varying abundances (quantified by digital PCR and expressed in copies/mL; **Supplementary Data 3**) (O'Sullivan et al., 2021) were evaluated using the ONT 16S Barcoding all-in-one kits and two different sequencing approaches, the ONT MinION flow cell and the Flongle flow cell, to assess assay performance and detection capabilities. Besides accuracy, factors such as ease of use, turnaround time for results, and cost were also reviewed. For consistency in this study, bioinformatics analysis was performed using BugSeq 16S analysis (v5.0) for all datasets. The results were expressed in average RA (%) of bacterial species detected in the MCM2α and MCM2β materials compared to AA (%) provided by the manufacturers (**Supplementary Data 5**). The AA of each species was expressed as a percentage of the overall copy number defined by the dPCR. The nominal abundance (theoretical or expected species abundance expressed in copies/mL) of bacterial species in the MCM2 materials is also shown (**Figures 1**, **2**). To compare the similarity of the RA to the AA, a Bray Curtis Dissimilarity statistical measure was used to calculate the four key reporting measures for the MCM2α/β reagents (**Table 1**).

**Table 1:** Bray Curtis dissimilarity statistical measure for the MCM2α/β reagents using R9 chemistry.

	Actual Abundance	Relative Abundance			
**Sensitivity**	MCM2α/β	MCM2α MinION flow cell R9	MCM2α Flongle flow cell R9	MCM2β MinION flow cell R9	MCM2β Flongle flow cell R9
**Sensitivity**	100.00	93%	85%	100%	93%
**FPRA**	0.00	0.00 %	0.00 %	0.00 %	0.00 %
**Diversity**	16.00	13	12	15	14
**Similarity**	100%	71%	71%	83%	83%

The RA of bacterial organisms in the MCM2α/β were mostly consistent (CV<1) between replicates when sequenced using the MinION flow cell sequencing approach (R9 chemistry). *M. tuberculosis* was not detected in any of the three dilutions in MCM2α reagent nor was it detected in the 1:100 dilution of MCM2β; AA 0.07% and 11.95%, respectively. All other bacterial organisms within the MCM2β reagent were detected by the MinION flow cell sequencing approach (R9 chemistry). Statistically significant differences between replicates and the AA were observed for *S. enterica* (MCM2α neat, *p* = 0.04, Kruskal-Wallis) and *H. influenzae* (MCM2α 1:10 dilution, *p* = 0.04, Kruskal-Wallis). A summary of these findings is presented in **Table 2**.

**Table 2:** Detection and consistency of bacterial organisms in MCM2α/β reagents using MinION flow cell and Flongle flow cell sequencing approaches (R9 chemistry).

Organism	Reagent	Dilution	MinION Flow Cell (R9) Detection	Flongle Flow Cell (R9) Detection
***M. tuberculosis***	MCM2α	All dilutions	Not detected	Not detected
	MCM2β	All dilutions	Neat and 1:10 detected, 1:100 Not detected	Not detected
***A. baumannii***	MCM2α	All dilutions	Detected (consistent); *p >* 0.05 (Kruskal Wallis)	Not detected
***N. meningitidis***	MCM2β	All dilutions	Detected (consistent); *p >* 0.05 (Kruskal Wallis)	Not detected
***S. enterica***	MCM2α	Neat	Detected (inconsistent) *p =* 0.04 (Kruskal Wallis)	Not reported
***H. influenzae***	MCM2α	1:10 dilution	Detected (inconsistent) *p =* 0.04 (Kruskal Wallis)	Not reported
***E. coli***	MCM2β	1:100 dilution	Detected (consistent); *p >* 0.05 (Kruskal Wallis)	Detected (inconsistent) *p =* 0.04 (Kruskal Wallis)
***S. pneumoniae***	MCM2β	1:100 dilution	Detected (consistent); *p >* 0.05 (Kruskal Wallis)	Detected (inconsistent); *p =* 0.02 (Kruskal Wallis)
***General Findings***	MCM2α/β	All dilutions	Mostly Consistent; *p >* 0.05	Inconsistent; Flongle flow cell struggles with low-abundance organisms

*p-value: Statistical significance determined using the Kruskal-Wallis test for comparisons between replicates: *p*<0.05 indicates statistically significant difference

The RA of bacterial organisms in the MCM2α/β materials was largely consistent between replicates (CV< 1) when sequenced using the Flongle flow cell sequencing approach (R9 chemistry). However, a notable finding was that the lowest abundant organisms (AA< 1%) in the MCM2α/β materials, particularly in the 1:100 dilution, were not detected using the Flongle flow cell sequencing approach (R9 chemistry). Statistically significant differences between replicates were observed for *E. coli* (MCM2β 1:100 dilution, *p* = 0.04, Kruskal-Wallis) and *S. pneumoniae* (MCM2β 1:100 dilution, *p* = 0.02, Kruskal-Wallis). Neither *A. baumannii* nor *M. tuberculosis* were detected in any of the three dilutions of MCM2α or MCM2β materials using the Flongle flow cell sequencing approach (R9 chemistry). Similarly, *N. meningitidis* was not detected in any of the three dilutions of the MCM2β material. Additionally, certain organisms, including *S. enterica*, *S. epidermidis*, *E. faecalis*, *E. faecium*, *P. aeruginosa*, *S. aureus*, and *S. pneumoniae*, were not detected in at least one dilution series. A summary of these findings is provided in **Table 2**.

#### MinION flow cell demonstrated higher accuracy compared to Flongle flow cell

To compare the performance of the new R10.4.1 MinION flow cell and Flongle flow cell and version 14 kit chemistry, Bray Curtis dissimilarity measure was used to compare the RA of microbes in the neat preparation of the MCM2α/β reagents to the AA and to the RA obtained using the MinION flow cell and Flongle flow cell and original R9 kit chemistry (**Table 3**). For the R10.4.1 Flongle flow cell, the sensitivity was 75% which is less than the R9 chemistry which has a sensitivity of 85%. *A. baumannii, E. coli, M. tuberculosis, P aeruginosa*, and *S. pneumonia*e were not detected by the R10.4.1 Flongle flow cell and v14 kit chemistry sequencing approach. For the R10.4.1 Flongle flow cell sequencing approach the sensitivity dropped to 74% compared with 93% Sensitivity attributed to the R9 MinION flow cell chemistry. *A. baumannii, M tuberculosis, N. meningitidis* and *S. epidermidis* were not detected by the R10.4.1 MinION flow cell chemistry and corresponding v14 kit version.

**Table 3:** Bray Curtis dissimilarity statistical measure for the MCM2α/β reagents using R10.4.1 chemistry.

	Actual Abundance	Relative Abundance			
Sensitivity	MCM2α/β	MCM2α MinION flow cell R10.4.1	MCM2α Flongle flow cell R10.4.1	MCM2β MinION flow cell R10.4.1	MCM2β Flongle flow cell R10.4.1
**Sensitivity**	100.00	93%	75%	100%	74%
**FPRA**	0.00	0.00 %	0.00 %	0.00 %	0.00 %
**Diversity**	16.00	13	9	15	11
**Similarity**	100%	71%	71%	83%	83%

The relative abundance (RA) of organisms in the MCM2α reagent showed a similarity of 71% to the absolute abundance (AA) for both R9 and R10.4.1 MinION flow cell versions. Overall, there was a 94% similarity in the RA of microbes in MCM2α across both MinION flow cell chemistries. For the MCM2β reagent, the RA demonstrated an 83% similarity to AA for both R9 and R10.4.1 MinION flow cell versions, with an overall similarity of 92%. In the R10.4.1 Flongle flow cell, the RA for MCM2α showed a similarity of 69% to AA, slightly lower than the 71% for the R9 Flongle flow cell. Overall similarity in MCM2α across both Flongle flow cell versions was 98%. The MCM2β reagent displayed an 83% similarity to AA for both R9 and R10.4.1 Flongle flow cell versions, with an overall similarity of 95%. These results highlight that both R9 and R10.4.1 MinION flow cell and Flongle flow cell versions, along with the old and updated version 14 of the ONT 16S kit, perform similarly when tested with standardized control materials, underscoring the importance of using such controls for revalidation. Based on BugSeq (v5.0) fully automated bioinformatic analysis platform, there was no evidence of cross-contamination during library preparation for all sequencing experiments, with zero read count in all three controls after read-clustering and filtering (minimum cluster size threshold of 30 reads).

#### Performance of in-house developed 16S ONT RBK method

**Figure 3:**
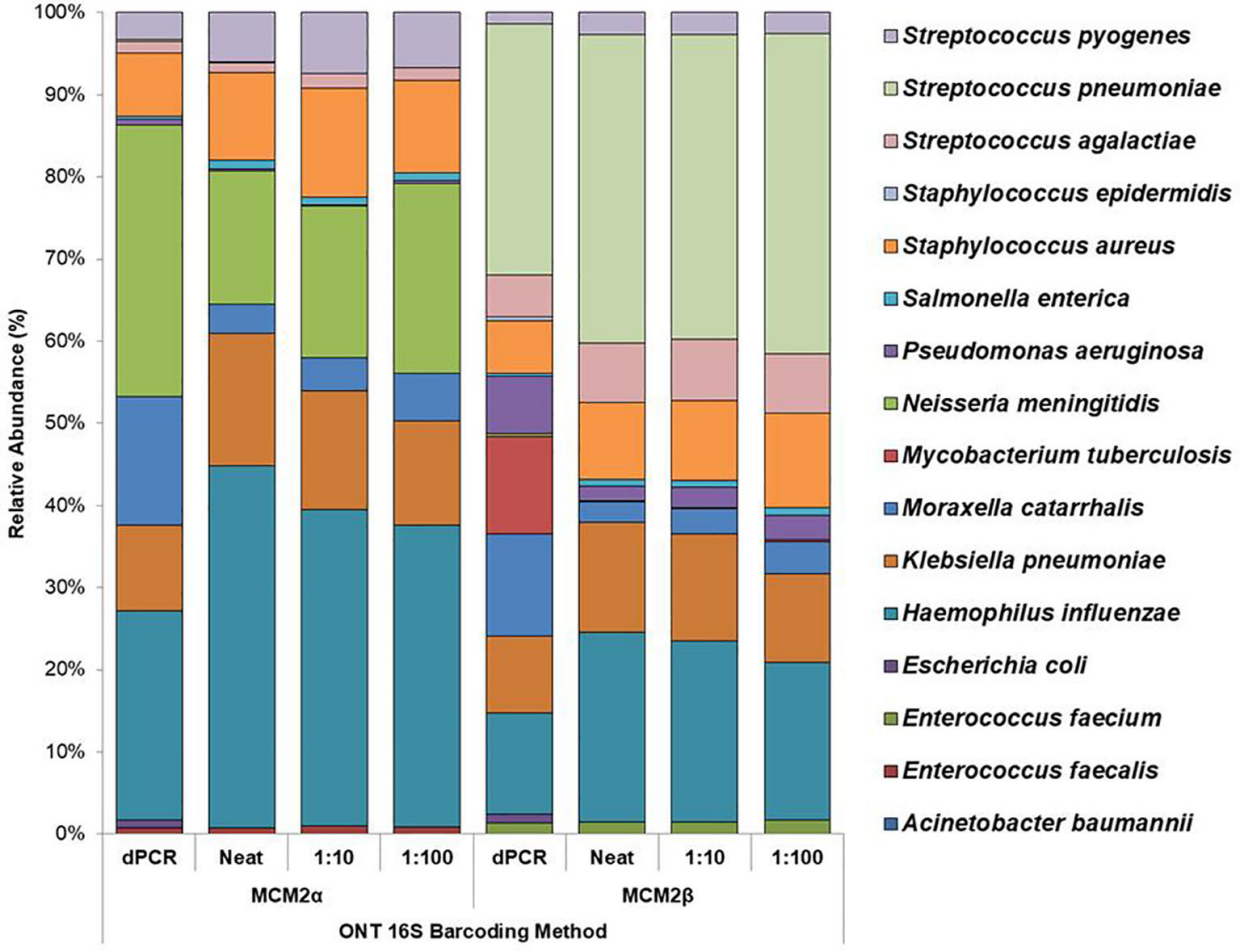
Average relative abundance (%) of bacterial organisms present in MCM2α and MCM2β obtained by ONT sequencing using the R10.4.1 MinION flow cell and ONT 16S Barcoding all-in-one kit (v14). Three concentrations of the MCM2α and MCM2β materials were tested in triplicate (neat, 1:10 and 1:100) and analysis by EPI2ME Desktop agent. The species abundance (%) of each dilution is reported, including the dPCR reported composition for the two materials (**Supplementary Data 3**).

**Figure 4:**
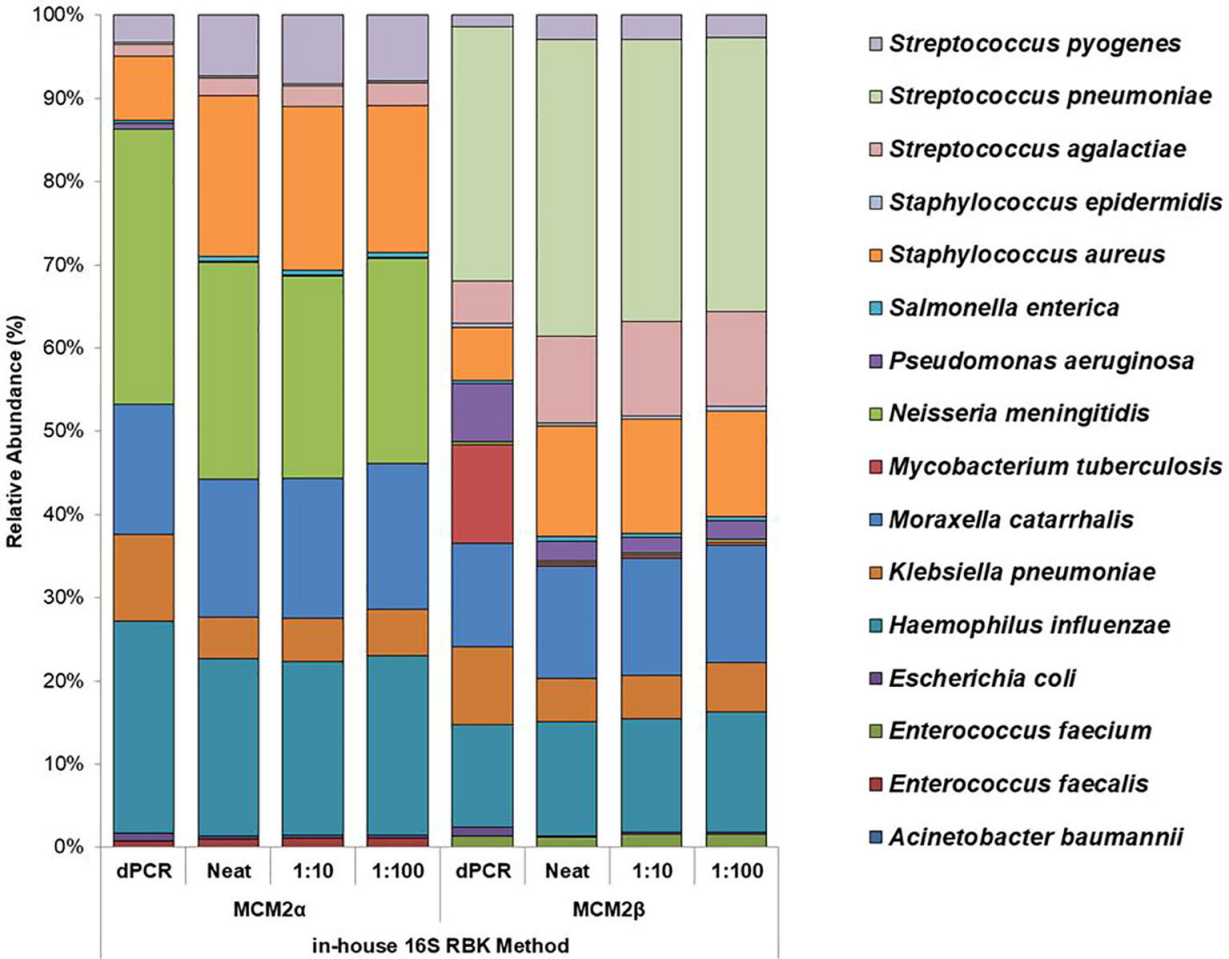
Average relative abundance (%) of bacterial organisms present in MCM2α and MCM2β obtained by ONT sequencing using the R10.4.1 MinION flow cell and the in-house developed 16S ONT RBK method. Three concentrations of the MCM2α and MCM2β materials were tested in triplicate (neat, 1:10 and 1:100) and amplified in two PCRs targeting the V1-V2 and V1-V9 genomic regions of the 16S rRNA gene; analysis by EPI2ME Desktop agent. The species abundance (%) of each dilution is reported, including the dPCR reported composition for the two materials (**Supplementary data 3**).

The performance of the in-house developed 16S ONT RBK method was compared against the ONT 16S Barcoding all-in-one kit (v14) using MCM2α/β materials and results analysed using EPI2ME Desktop Agent (**Figures 3**, **4**). Sequencing data was analysed with the ONT EPI2ME Desktop agent to compare performance. The in-house developed ONT RBK method outperformed the ONT 16S Barcoding all-in-one kit in detecting bacterial organisms within the MCM2α/β materials. All organisms in MCM2β were identified by the in-house RBK method. However, the ONT 16S Barcoding all-in-one kit failed to detect *E. coli* and *A. baumannii* in all MCM2α replicates and did not detect *E. coli, S. epidermidis, N. meningitidis*, and *A. baumannii* in MCM2β. *M. tuberculosis* was not detected by either method in MCM2α. The EPI2ME Desktop Agent bioinformatics pipeline produced speciation errors, misidentifying *N. meningitidis* and *E. coli* as closely related species *N. cinerea and E. furgusonii*, respectively. For the purposes of this analysis, these misidentified species were included in the overall relative abundance calculations.

### Effect of DNA extraction method on DNA yield, amplification, and sequencing

To determine extraction efficiency, the WHO WC-Gut RR, containing bacterial cells from twenty bacterial species in equal ratios, was extracted neat and in triplicate using four different extraction methods and eluted into a final elution volume of 50-60µL. The WHO DNA-Gut-Mix RR was sequenced alongside the WHO WC-Gut RR to determine whether bias comes from the extraction process or the sequencing and bioinformatics analysis, as the two reagents are equivalent.

When comparing the DNA extraction yield of the neat WHO WC-Gut-RR material quantified by Qubit (total ng per extract) from the four methods, the QIAGEN EZ2 DNA/Tissue kit yielded the highest average concentration of genomic DNA at 881.6ng. Qiagen EZ2 Virus Mini had a DNA yield of 302.4ng, AusDiagnostics MT-Prep Pathogen had a DNA yield of 361ng and the Qiagen QIAmp DNA/Blood had a DNA yield of 307ng. To determine statistically significant differences, a one-way ANOVA was performed, yielding an F-statistic of 97.45 and a p-value< 0.05, indicating significant differences in DNA yield across the four extraction methods. Tukey's Honestly Significant Difference (HSD) test was subsequently applied to identify specific differences between the extraction methods. A summary of the findings is presented in **Table 4**.

**Table 4:** Results of Tukey's HSD *post-hoc* test for DNA yield comparison across extraction methods.

Comparison	Mean Diff.	p-value	Statistically Significant Difference
***Aus Diagnostics MT-Prep vs Qiagen EZ1/2 DNA Tissue***	-2.17	<0.001	Yes
***Aus Diagnostics MT-Prep vs Qiagen EZ1/2 Virus Mini***	-15.23	<0.001	Yes
***Aus Diagnostics MT-Prep vs*** *QIAmp DNA/Blood kit*	0.69	0.695	No
***Qiagen EZ1/2 DNA Tissue vs Qiagen EZ1/2 Virus Mini***	-13.06	<0.001	Yes
***Qiagen EZ1/2 DNA Tissue vs*** *QIAmp DNA/Blood kit*	-1.48	0.363	No
***Qiagen EZ1/2 Virus Mini vs*** *QIAmp DNA/Blood kit*	11.58	<0.001	Yes

The table presents the pairwise comparisons of DNA yield (ng) between four DNA extraction methods. The Mean Diff. represents the difference in means between each pair of methods, with the corresponding p-value indicating whether the difference is statistically significant (p< 0.05). Statistically significant differences are marked with a "Yes."

The nucleic acid yield was further evaluated using dPCR, targeting both the 16S rRNA gene and the *E. coli uidA* gene. DNA extracts obtained from the QIAGEN Virus Mini and DNA tissue kits showed the greatest yield in terms of copies/µL according to dPCR (**Supplementary Data 3**). Neat DNA extracts of the WHO WC-Gut RR material were prepared for sequencing using the ONT 16S Barcoding all-in-one kit (v14) and run on an R10.4.1 MinION flow cell. Sodium azide present in the elution buffer of the Qiagen EZ2 extraction kits (Virus mini and DNA Tissue kits) was found to be incompatible with ONT sequencing as all sequencing experiments using DNA extracts generated from these extraction kits had no read data when analysed by BugSeq (v5.0) bioinformatic pipeline. This finding was found on multiple repeat experiments and has also described by elsewhere in the literature (Eagle et al., 2023). DNA extraction was successfully repeated by performing a bead clean up using AMPure XP beads (1:1.8 ratio) to remove sodium azide before library preparation. Following sequencing of all the DNA extracts from the WHO WC-Gut RR material, the RA of the bacterial organisms present in the WHO WC-Gut RR material was compared to the ‘ground truth’ (Anwar et al., 2023).

Species-level identification was successfully achieved for all organisms present in the WHO Gut RR material (**Supplementary Data 6**). However, to evaluate DNA extraction efficiency, relative abundance (RA) analysis of bacterial organisms in the WHO Gut-WC RR material was conducted at the genus level. This approach mitigated bioinformatics challenges and the inherent limitations of 16S analysis in distinguishing closely related species, such as the misclassification of *E. coli* and *Blautia wexlerae* (**Supplementary Data 6**), thereby reducing bias in evaluating the extraction process. To compare the similarity of the RA to the AA, Bray Curtis dissimilarity statistical measure was used to calculate the four key reporting measures (**Table 5**). FPRA was 0% for all the extraction methods, indicating no false positive results. The sensitivity was found to be between 75% - 82.1% (optimal 100%) because Diversity was between 12 and 13 (optimal 16), meaning that no method was able to extract all the bacteria in the whole cell reagent. None of the extraction methods could extract *Alistipes finegoldii*, *Clostridium butyricum* or *Ruminococcus gauvreauii*. In addition, *Parabacteroides distasonis* was only extracted by the AusDiagnostics MT Prep extraction method. The four different extraction methods had varied extraction efficiency for the remaining twelve bacterial genera in the reagent illustrated by variation in the Similarity measure, ranging from 38% - 45%, when compared to the AA of the WHO Gut-WC RR material. RA analysis of the WHO DNA-Gut-Mix RR showed an FPRA of 0%, sensitivity 100%, where all bacterial organisms were detected (diversity 16/16) and a similarity of 51.4% when compared to the AA.

**Table 5:** Bray Curtis Dissimilarity statistical measure for the WHO WC-Gut-RR material using R10.4.1 MinION flow cell and ONT v14 kit chemistry.

	Actual Abundance	Relative Abundance			
**Sensitivity**	WHO WC-Gut RR	AusDiagnostics MT-Prep kit	Qiagen QIAmp Blood/Tissue kit	Qiagen EZ2 DNA Tissue kit	Qiagen EZ2 Virus Mini kit
**Sensitivity**	100.00	81%	75%	75%	75 %
**FPRA**	0.00	0.00 %	0.00 %	0.00 %	0.00 %
**Diversity**	16.00	13	12	12	12
**Similarity**	100%	43%	45%	40%	38%

## Clinical application of the method

To evaluate the effectiveness of this approach for clinical use, thirty-four patient samples were tested in this study, including twelve 16S PCR-negative and twenty-two 16S PCR-positive samples. These samples previously underwent 16S PCR and conventional Sanger sequencing as part of routine diagnostic testing, in accordance with local protocols (Lampejo et al., 2021). Among the twenty-two 16S PCR-positive samples, seventeen had yielded a taxonomic identification of a bacterial organism. However, the remaining five positive samples did not provide taxonomic identification due to poor sequence quality, displaying a mixed electropherogram pattern that suggests a potential polymicrobial composition. To determine whether the proposed ONT approach could enhance the detection of bacterial pathogens and improve the characterization of polymicrobial infections, these thirty-four clinical samples were evaluated by comparing the performance of two different ONT library preparation protocols: the ONT 16S Barcoding all-in-one kit (v14) (SQK-16S114.24) and our in-house developed method using the ONT Rapid Barcoding kit (SQK-RBK-114.96), referred to as the "in-house developed ONT RBK method." All PCR amplicons were sequenced, and taxonomic identification results were compared to those obtained with the routine diagnostic method (16S rRNA gene PCR and Sanger sequencing). Only ten out of the thirty-four clinical samples produced comparable results across all three methods. The in-house developed ONT RBK method emerged as the most sensitive sequencing approach, successfully confirming the taxonomic identification of all twenty-four previously reported 16S PCR-positive clinical samples, while also identifying pathogenic bacteria in the twelve samples previously reported as 16S PCR-negative (**Supplementary Data 7**).

## Discussion

This study assessed the performance of 16S rRNA gene amplicon sequencing using ONT to detect clinically significant bacterial pathogens in clinical samples. Two mock microbial community samples (Metagenomic Control Materials MCM2α and MCM2β) were used to assess the performance, accuracy, ease of use and suitability of two different ONT sequencing approaches and library preparation kits for routine clinical diagnostics (**Table 1** and **3**). ONT MinION flow cell performed better than the Flongle flow cell and the in-house developed ONT RBK method performed better than the ONT 16S Barcoding all-in-one kit for the accurate detection and characterization of clinically relevant bacterial organisms, especially those in low abundance (**Figures 3**, **4**).

Using the well characterised MCM2α/β materials as a control, this study highlights the superior performance of the in-house developed ONT RBK method compared to the ONT 16S Barcoding all-in-one kit in detecting clinical pathogens at low abundance. The in-house RBK method detected all organisms in all MCM2β replicates whereas the ONT 16S Barcoding all-in-one kit failed to detect *E. coli* and *A. baumannii* in MCM2α and did not detect *E. coli, S. epidermidis, N. meningitidis*, and *A. baumannii* in MCM2β. Critically, *M. tuberculosis* was not detected in the MCM2α regardless of the sequencing approach or method used and was significantly underrepresented in the MCM2β material; AA 0.07% and 11.35%, respectively. The underrepresentation of *M. tuberculosis* in MCM2α may stem from its low abundance in the material. For MCM2β, this could result from multiple factors. Notably, *M. tuberculosis* has only one copy of the 16S rRNA gene in its genome (Watanabe et al., 2016; Valdivia-Anistro et al., 2016), and PCR-based bacterial detection is biased toward organisms with higher gene copy numbers particularly in mixed template material (Krehenwinkel et al., 2017). Variations in 16S rRNA gene copy number across bacteria can introduce amplification biases, highlighting the limitations of this molecular method (Louca et al., 2018). Additionally, the MCM2 materials are complex, polymicrobial reference samples, where low-abundance organisms with low copy numbers may be overshadowed by more abundant organisms with higher copy numbers during amplification. However, testing commercial DNA controls containing *M. tuberculosis* and known clinical samples positive for *M. tuberculosis* using the in-house ONT RBK method successfully detected the organism (data not shown), ruling out primer specificity as a major contributing factor. This study also highlighted that the MinION flow cell sequencing approach performs better than the Flongle flow cell approach for detecting organisms in low abundance (**Figures 1**, **2**). The underrepresentation of low-abundance organisms by the Flongle flow cell is likely due to the shorter sequencing time (5 hours vs. 18 for MinION flow cell), and fewer nanopores (50–60 vs. 800–1200 for MinION flow cell). This study found that the MinION flow cell generated 10–30 times more reads than the Flongle flow cell for the MCM2 reference materials, primarily due to its greater pore capacity and extended sequencing time (**Supplementary Data 8**). Further research is required to optimize sequencing durations on the MinION flow cell with the 16S Barcoding All-in-One Kit to establish its detection limits for low-abundance organisms. However, this was not explored in the current study, as the in-house ONT Rapid Barcoding Kit (RBK) method was identified as the superior sequencing protocol and prioritized for further development, with the goal of integrating it into routine diagnostic services. In the clinical sample study, a sequencing time of 12 hours was used, as recommended by the manufacturer for their Rapid Barcoding Kit. This duration enabled accurate species identification and demonstrated superior performance compared to both Sanger sequencing and the ONT 16S Barcoding All-in-One Kit. Given these advantages, future applications of the ONT Rapid Barcoding Kit will continue to adopt the 12-hour sequencing time. This clinically appropriate timeframe allows for overnight sequencing runs with results available the next day, significantly reducing turnaround times from 3–4 days with Sanger sequencing to 1–2 days with ONT.

Besides the bias detected in amplification and sequencing, a limiting factor in method accuracy is the DNA extraction. The efficiency of a DNA extraction method is not only judged based on the DNA yield and amplification specificity, but it is important to also assess its ability to successfully extract microbes evenly, especially clinically relevant pathogens.

To explore the influence of DNA extraction methods on the identification of clinically relevant pathogens, four extraction methods were evaluated using the WHO WC-Gut RR reference material. This material, consisting of 20 organisms each at an equal abundance of 5%, was specifically designed to minimize variability and provide a reliable basis for assessing extraction bias. While the organisms were primarily analyzed at the genus level to mitigate bioinformatics and database limitations—such as the misclassification of closely related species like *E. coli* and *Blautia wexlerae*—species-level identification was successfully achieved for all organisms in the reference material (**Supplementary Data 6**). This highlights the dual utility of the reference material: it not only facilitates evaluation of extraction efficiency but also underscores the potential for refining bioinformatics workflows to overcome species-resolution challenges. These findings emphasize the importance of optimizing both laboratory methods and computational tools to ensure accurate pathogen identification. Results from this study showed that the QIAGEN EZ2 DNA Tissue extraction kit yielded the highest concentration of nucleic acid which subsequently allowed for greater amplification efficiency as shown by dPCR targeting *uidA* and 16S rRNA genes (**Supplementary Data 3**). In addition, high variability was observed in the Bray Curtis dissimilarity measure (38-45%), indicating the level of bias in the extraction methods when compared to the ‘ground truth’ (**Table 5**). Parabacteroides spp. was exclusively extracted using the AusDiagnostics MT-Prep method, indicating potential challenges in DNA extraction for this organism when using Qiagen extraction methods. Ruminococcus spp.*, C. butyricum* and *A. finegoldii* were not extracted by any of the extraction methods in the WC-Gut-Mix RR but were detected in the sequencing control (DNA-Gut-Mix RR), suggesting that they were lost during the DNA extraction process. *C. butyricum* is known to be an exceedingly difficult organism to extract even from pure cultures (Esteban et al., 2020; Sergaki et al., 2022). In addition, *A. finegoldii* is a small circular bacterium which may avoid cellular disruption during the bead beating process and therefore likely difficult to extract as previously described (Sergaki et al., 2022). Research has demonstrated that the detection rates of organisms can vary depending on the duration of lysis by bead beating, with some organisms requiring minimal (0-1 min) or extensive (4-9 min) bead-beating for optimal recovery (Zhang et al., 2021). *C. butyricum* and *A. finegoldii* are good examples of microbes that are difficult to extract in the context of multi microbial communities and it is important to include these organisms in standardization studies to highlight the limitations of DNA extraction methodologies. The DNA extraction methods used in this study were chosen for their widespread use in UK clinical laboratories and their compatibility with in-house extraction platforms. These kits are designed to extract DNA from complex human tissue samples, making them suitable for validating molecular methodologies intended for clinical applications. While other kits specifically developed for bacterial DNA extraction may offer superior performance, further studies are needed to evaluate their efficacy. Such kits would require in-house validation to ensure their suitability for extracting bacterial DNA from complex clinical samples. Additionally, they must be compatible with automated extraction platforms to integrate seamlessly into the workflow of busy diagnostic laboratories.

In the clinical sample study, the in-house developed ONT RBK method demonstrated superior sensitivity in detecting bacterial organisms from clinical samples that were previously culture-negative and 16S PCR-negative. This method employs two distinct PCR primer pairs targeting the V1-V2 and V1-V9 regions of the 16S rRNA gene, enhancing the range of detectable bacterial species. The primer sets were carefully selected for their ability to provide broad bacterial coverage while minimizing amplification bias, as supported by established literature (Harris and Hartley, 2003; Dos Santos et al., 2019). The PCR protocol was optimized to achieve robust amplification from clinical samples while maintaining specificity. By incorporating two primer sets, this method broadens detection coverage and ensures the identification of a wide array of clinically relevant pathogens. The dual-primer approach mitigates amplification biases associated with single primer sets and improves overall sensitivity. Additionally, the complementary nature of the primer sets provides a safeguard; when species-specific polymorphisms in conserved target regions hinder the annealing of one set, the other set compensates effectively, ensuring reliable detection.

A key consideration in amplicon-based 16S rRNA gene sequencing is the presence of chimeric reads, which are artifact sequences formed when two or more template sequences incorrectly anneal during PCR. Chimeras are typically observed in samples with mixed templates, occurring at an estimated frequency of approximately 30% (Wang and Wang, 1996). However, in clinical diagnostics, particularly with samples derived from sterile anatomical sites devoid of background flora, the frequency of chimeric reads is generally much lower. To ensure the quality of the output, the BugSeq (v5.0) 16S pipeline requires both 80% query coverage and 80% sequence identity for the classification of consensus sequences (Jung and Chorlton, 2021). This stringent threshold significantly reduces the occurrence of false positive classifications due to chimeras, as these artifacts typically fail to align at such a high percentage against a single reference sequence. Additionally, BugSeq filters out reads shorter than 1000 bp or longer than 1850 bp during preprocessing, effectively excluding chimeras or artifacts that would otherwise exhibit abnormal read lengths. The low fraction of unclassified and filtered reads confirms that the rate of chimeras/artifacts in these samples is negligible. Therefore, the proportion of chimeric reads was not quantified in this study. At present, no validated bioinformatics tools exist for detecting chimeras in Nanopore amplicon data, indicating an area for future research aimed at developing and validating such tools for long-read sequencing data. Nevertheless, the accurate identification of organisms in standardized reference materials and known positive clinical samples in this study provides clinical confidence that any chimeric reads present did not affect species-level resolution. Future studies should incorporate the assessment of chimeric read frequency to further validate the accuracy of these analyses, utilizing validated bioinformatics tools specifically designed for long-read sequencing data. To further assess the quality of the sequencing output, a representative subset of positive clinical samples was aligned to reference genomes using the ONT EPI2ME platform ‘Alignment’ workflow, with reference genomes obtained from the NCBI database. The alignment results confirmed species-level identification, demonstrating complete coverage of the 16S rRNA gene and excellent sequence depth for the analyzed subset. These findings further validate the robustness of the method (**Supplementary Data 4A–D**).

Taxonomic identification using the in-house developed ONT RBK method uncovered clinically meaningful results that were missed by the current Sanger sequencing method and the ONT 16S Barcoding all-in-one kit (**Supplementary Data 7**). The improved sensitivity of the in-house developed ONT RBK method was particularly noticeable in pus, joint fluid and heart valve samples, which were culture negative (Samples 1-12, **Supplementary Data 7**). Assessing read count of organisms detected in clinical samples was not a primary focus of this study, as it can vary due to stochastic effects. In the context of 16S rRNA gene sequencing using ONT for clinical diagnostics, the primary goal is qualitative detection of pathogens in sterile anatomical site samples. In such cases, read count is less critical for determining the clinical significance of the findings. The results of 16S amplicon-based sequencing for these samples have been used to formulate a definitive diagnosis due to organisms being uncultivable by routine culture. Consequently, taxonomic identification using the in-house developed ONT RBK method led to the amendment of seventeen patient reports which were previously reported as ‘Bacterial DNA NOT detected’ All clinical results were evaluated within the clinical context and alongside other microbiological findings to determine their significance. Samples were obtained from sterile anatomical sites devoid of commensal flora, where pathogen identification strongly suggests a probable cause of the patient’s pathology. The resolution of symptoms following targeted antimicrobial therapy further supports a cause-and-effect relationship between the identified organisms and the infectious pathology. In addition, the use of well-characterized controls validated the assay’s sensitivity and specificity, enabling reliable detection of bacterial organisms across varying abundance. Interpretation of 16S assays—whether using Sanger, Illumina, or Nanopore sequencing—requires a multidisciplinary approach, incorporating infection markers, prior microbiological findings, clinical presentation, histopathology (if applicable), and clinical suspicion. Ultimately, the observed symptom resolution highlights the assay's clinical utility and its potential to enhance patient outcomes. These findings directly influenced patient management and led to improved clinical outcomes.

The performance of ONT 16S Barcoding all-in-one kit on clinical samples was found to be sub-optimal, as it failed to identify 9/17 bacterial organisms from clinical samples. While this method performed relatively well with the standardised materials, which are in high cell concentrations, it did not perform as well with clinical samples containing very low bacterial yields (<1 CFU). As seen in the MCM2α/β experiment, the ONT 16S Barcoding all-in-one kit performed sub-optimally in detecting low abundant organisms especially at lower dilutions and when using the Flongle flow cell sequencing approach (R9 and 10.4.1 chemistries) (**Figures 1**, **2**).

Results from this study highlight the importance of several factors that must be considered if ONT is to be implemented into diagnostic microbiology laboratories for the diagnosis of culture negative infections. Frequent changes to ONT kit versions, sequencing chemistry and EPI2ME bioinformatic pipelines highlight the importance of validation and re-validation to respond to the ever-evolving landscape of sequencing technologies. Development of assured and metrologically defined reference materials are essential so that re-validation can be performed promptly, ensuring that the diagnostic service is not impacted and to maintain high quality standards for successful accreditation. A significant limitation to implementing advanced sequencing approaches like ONT in NHS diagnostic laboratories is the lack of bioinformatics resources. The bioinformatics pipelines used in this study demonstrate potential solutions for laboratories without in-house bioinformaticians. The BugSeq online platform was highly user-friendly, though it incurs a cost. ONT’s EPI2ME platform is also accessible and user-friendly but requires high-performance computing and bioinformatics expertise for initial setup and optimization, though the software itself is free. Other pipelines, such as Emu, necessitate bioinformatics expertise for command-line functionality (Curry et al., 2022). Additionally, bacterial speciation is influenced not only by primer selection but also by the database used for analysis and so variation in databases used can lead to variation in results. Consequently, variations in databases must be carefully validated to ensure the accuracy of results. A major limitation of the 16S rRNA gene sequencing method in clinical practice is that, for some species, the sequence may be ambiguous, complicating the differentiation between closely related, clinically significant species or phenotypes (e.g., Escherichia and Shigella spp.; **Supplementary Data 6**) (Clarridge, 2004). This challenge arises from microheterogeneity within the 16S rRNA gene of certain genera and must be carefully considered when analyzing 16S sequencing data. All results should be interpreted in the context of the clinical scenario and any prior microbiological investigations and discussed within the clinical team before making decisions regarding changes to patient management. Findings from this study highlight the critical factors required for the successful implementation of 16S amplicon-based sequencing using ONT in a diagnostic microbiology laboratory. Key considerations include primer selection, sequencing approach, library preparation kit, sequencing duration, and database choice, all of which significantly influence sensitivity and overall performance.

## Conclusions

This study demonstrates that an in-house developed ONT method utilizing the ONT Rapid Barcoding kit and R10.4.1 MinION flow cell is the best approach for diagnosing culture-negative infections from sterile anatomical sites, outperforming the ONT 16S Barcoding all-in-one kit and traditional Sanger sequencing.

This is the first study to provide a robust validation protocol for 16S amplicon-based sequencing using ONT, providing a comparative analysis of various sequencing approaches and assay chemistries, emphasizing the critical need for laboratory standardization in diagnostic techniques; particularly regarding the biases introduced during sample processing, DNA extraction, amplification, and sequencing. In addition to ensuring continuous quality assurance, the use of well characterized reference materials is crucial for validating emerging diagnostic technologies and establishing a framework for implementing ONT sequencing protocols in clinical microbiology laboratories. This study establishes a foundation for advancing diagnostic microbiology and improving patient care outcomes through enhanced detection capabilities. It provides essential insights for UK agencies involved in External Quality Assurance (EQA) materials, such as UK NEQAS, and supports the licensing of new diagnostics by the MHRA, as well as the review and accreditation of diagnostic laboratories by UKAS. Ultimately, these efforts will facilitate the integration of innovative methodologies into routine diagnostic practices on a broader scale.

## Data Availability

The original contributions presented in the study are included in the article/**Supplementary Material**. Further inquiries can be directed to the corresponding author.
