# Correction: Standardization of 16S rRNA gene sequencing using nanopore long read sequencing technology for clinical diagnosis of culture negative infections

**DOI:** 10.3389/fcimb.2026.1799339

**Published:** 2026-02-13

**Authors:** Ian Butler, Olivia Turner, Kulsoom Mohammed, Mazeda Akhtar, Daniel Evans, Jonathan Lambourne, Kathryn Harris, Denise M. O’Sullivan, Chrysi Sergaki

**Affiliations:** 1 Department of Microbiology, National Health Services (NHS) East and South East London Pathology Partnership, Bart’s Health NHS Trust, London, United Kingdom; 2 Faculty of Biology, Medicine and Health, The University of Manchester, Manchester, United Kingdom; 3 Molecular and Cell Biology, National Measurement Laboratory (NML), Laboratory of the Government Chemist (LGC), London, United Kingdom; 4 School of Biosciences & Medicine, University of Surrey, Guildford, United Kingdom; 5 Medicines and Healthcare Products Regulatory Agency (MHRA), London, United Kingdom

**Keywords:** 16S rRNA, Oxford nanopore, long read sequencing, standardization, culture negative

Affiliation “^5^Faculty of Biology, Medicine and Health, The University of Manchester” was omitted for author Ian Butler. This affiliation has now been added for author Ian Butler

“Ian Butler^1,2*^ Olivia Turner^1^, Kulsoom Mohammed^1^, Mazeda Akhtar^1^, Daniel Evans^3^, Jonathan Lambourne^1^, Kathryn Harris^1^, Denise M. O’Sullivan^3,4^ and Chrysi Sergaki^5^

^1^Department of Microbiology, National Health Services (NHS) East and South East London Pathology Partnership, Bart’s Health NHS Trust, London, United Kingdom

^2^Faculty of Biology, Medicine and Health, The University of Manchester

^3^Molecular and Cell Biology, National Measurement Laboratory (NML), Laboratory of the Government Chemist (LGC), London, United Kingdom

^4^School of Biosciences & Medicine, University of Surrey, Guildford, United Kingdom

^5^Medicines and Healthcare Products Regulatory Agency (MHRA), London, United Kingdom”

In the **Acknowledgements**, “This work was supported by the National School of Healthcare Science (NSHCS) as part of the Higher Specialist Scientist Training (HSST) program, UK” was omitted in error.

The corrected Acknowledgments section appears below.

## Acknowledgments

This research was part of the Healthcare Scientist (HCS) Knowledge Transfer Partnership (KTP) programme in affiliation with the National Measurement System (NMS) organisations and the United Kingdom Accreditation Service (UKAS). Acknowledgement Rui Pereira at NML for his contributions to this research. Also, acknowledgements to Neil Almond and Paul Matejschuk from NIBSC and Lorraine Turner and Alyson Bryant from UKAS and everyone at the NMS who supported this research. Special thanks to Ruben Mertens and the Molecular Microbiology team at Bart’s Health NHS Trust and also to Josh Chorlton and Sam Chorlton at BugSeq for their bioinformatic analysis support. This work was supported by the National School of Healthcare Science (NSHCS) as part of the Higher Specialist Scientist Training (HSST) program, UK.”

The original version of this article has been updated.

