# Supplementary material for: Standardization of 16S rRNA gene sequencing using nanopore long read sequencing technology for clinical diagnosis of culture negative infections
Source: Front Cell Infect Microbiol. 2025 Mar 6;15:1517208. doi: 10.3389/fcimb.2025.1517208 (PMC11922894; doi:10.3389/fcimb.2025.1517208)
Supplement: Supplementary file 1 [file DataSheet1.docx]

Supplementary Data 1: Nominal relative abundance of the microbial composition in MCM2α/β materials.

Supplementary Data 2: Microbial composition of the WHO WC-Gut Reference Reagents (NIBSC 22/210), with each organism present at equimolar concentrations (5% relative abundance per organism).

Supplementary Data 3: Digital PCR (dPCR) results targeting the 16S rRNA gene and the E. coli target gene (uidA).

Supplementary Data 4a: Sequence alignment of Clinical Sample 2 (Streptococcus pyogenes, identified from joint fluid using the in-house ONT RBK method) performed with the EPI2ME platform's 'Alignment' workflow. The alignment showed 99–100% cumulative coverage across the full 1500 bp of the 16S rRNA gene, with a sequence depth of x4750..
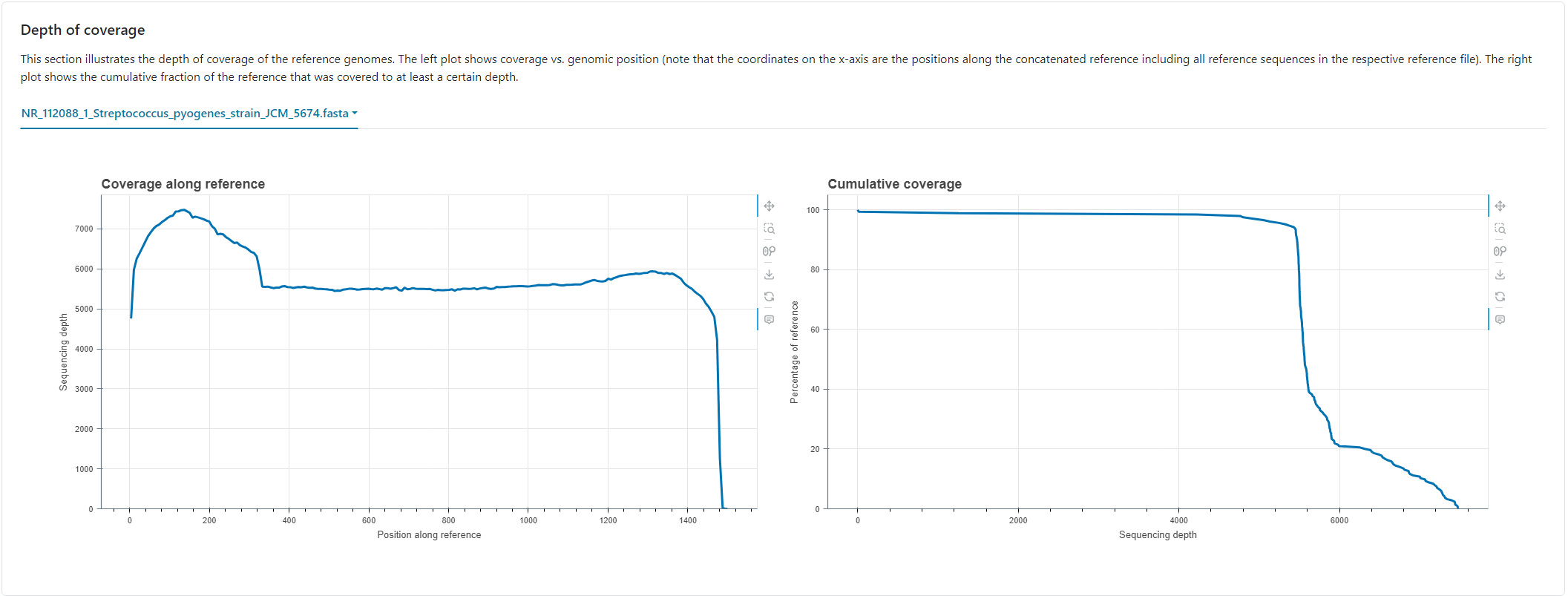


Supplementary Data 4b: Sequence alignment of Clinical Sample 27 (Staphylococcus roterodami, identified from a pus sample using the in-house ONT RBK method) performed with the EPI2ME platform's 'Alignment' workflow. The alignment showed 90–100% cumulative coverage across the full 1500 bp of the 16S rRNA gene at a sequence depth of x1750. As Staphylococcus roterodami is part of the Staphylococcus aureus complex, reads were aligned to the Staphylococcus aureus reference gene for comparison. This alignment indicated 90–100% cumulative coverage across the full 1500 bp of the gene at a sequence depth of x300, supporting the likely identification of Staphylococcus roterodami. However, it is reportable clinically as part of the Staphylococcus aureus complex.


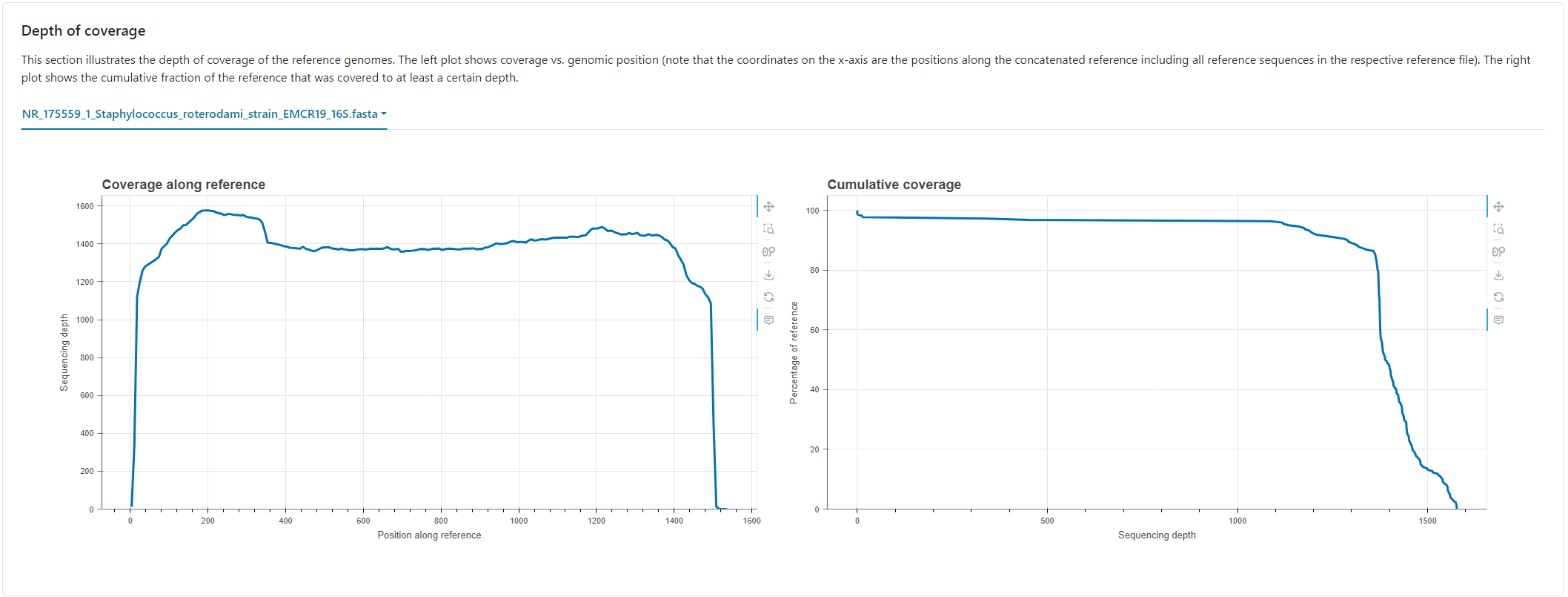


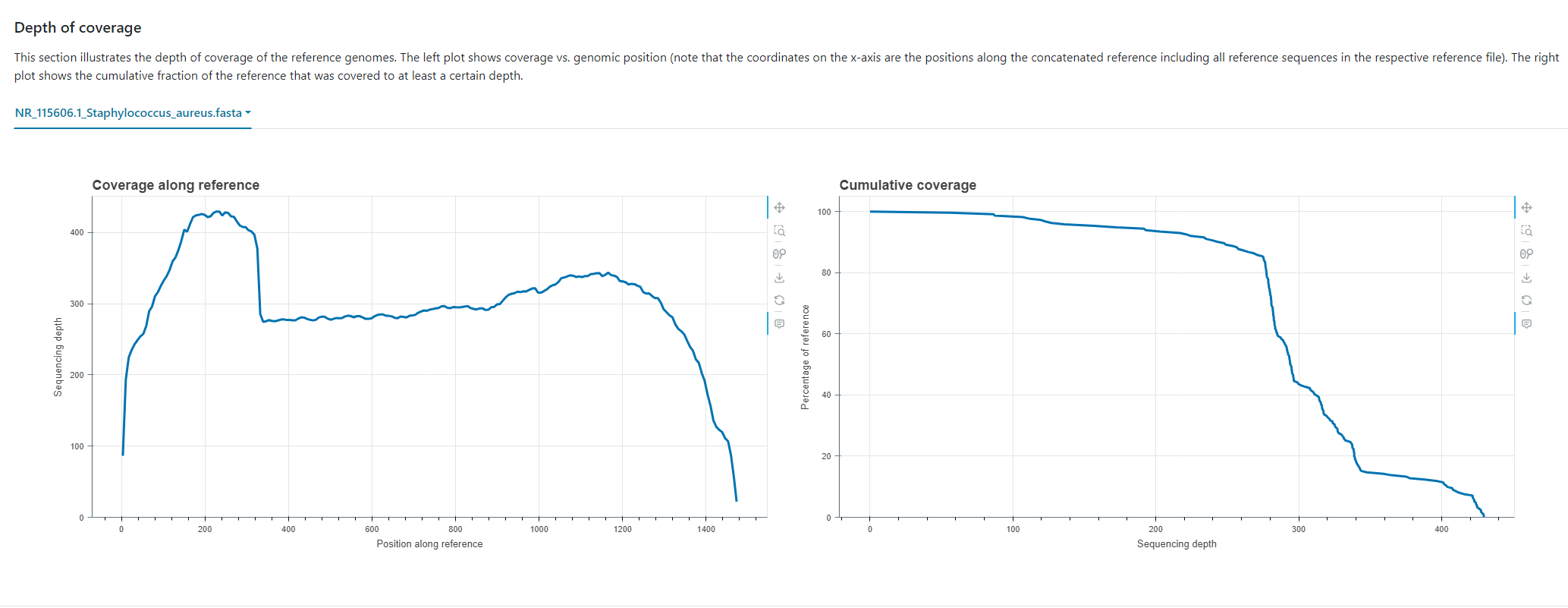


Supplementary Data 4c: Sequence alignment of Clinical Sample 25 (Streptococcus pneumoniae, identified from CSF using the in-house ONT RBK method) performed with the EPI2ME platform's 'Alignment' workflow. The alignment showed 90–100% cumulative coverage across the full 1500 bp of the 16S rRNA gene at a sequence depth of x2000.
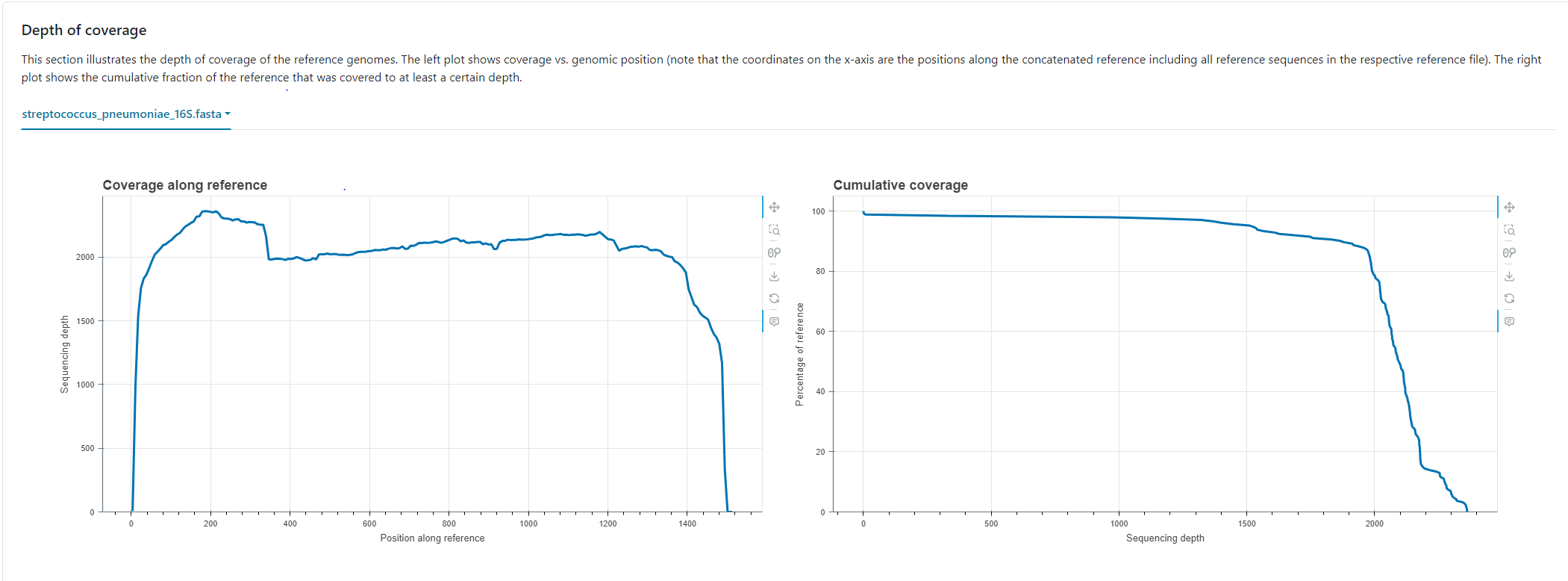


Supplementary Data 4d: Sequence alignment of Clinical Sample 29 (Staphylococcus aureus, identified from a tissue sample using the in-house ONT RBK method) performed with the EPI2ME platform's 'Alignment' workflow. The alignment showed 95–100% cumulative coverage across the full 1500 bp of the 16S rRNA gene at a sequence depth of x4000.
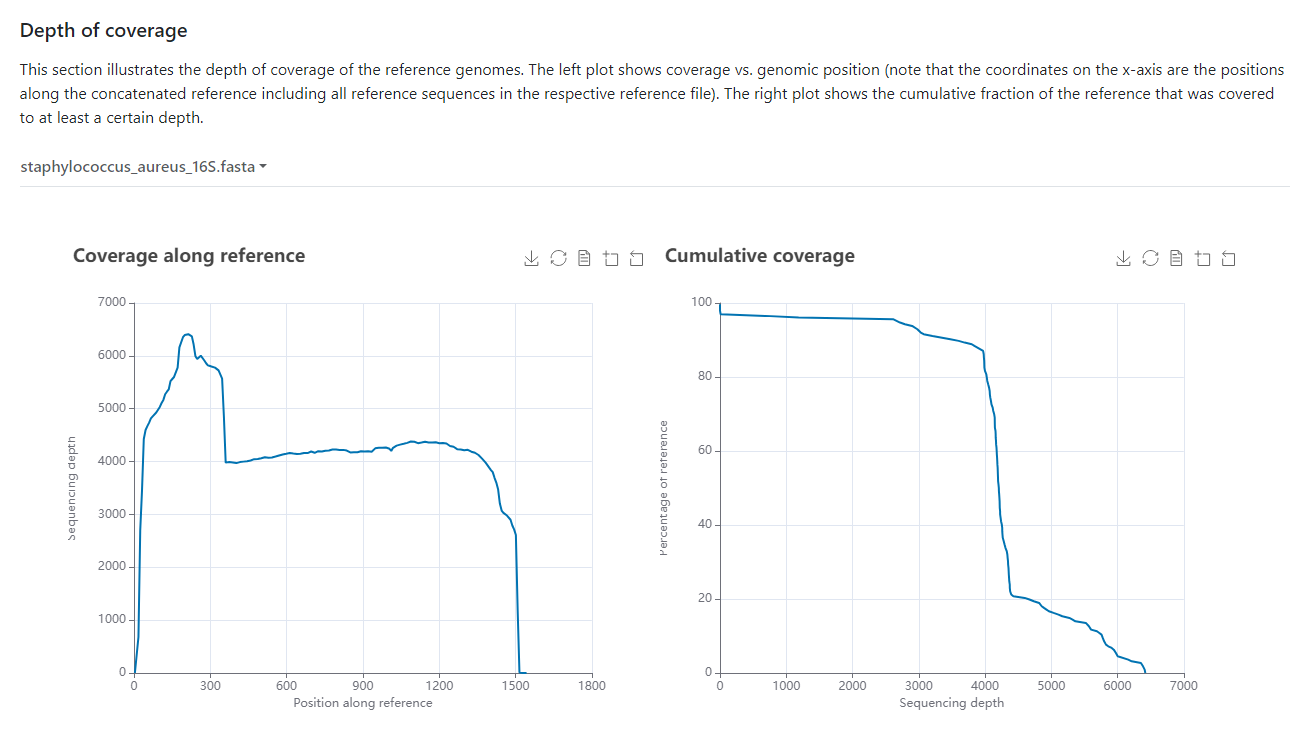
Supplementary Data 5: Microbial composition of MCM2α/β materials – actual abundance determined by dPCR and relative difference compared to nominal abundance.

Supplementary Data 6: Speciation of bacterial organisms in the WHO Gut RR materials using the in-house 16S ONT RBK method with the EPI2M platform and the 'wf-16S' workflow employing the minimap2 approach. Note the resolution challenges for closely related species (e.g., Escherichia/Shigella spp., Blautia spp; highlighted in orange) due to the inherent limitations of 16S rRNA gene sequencing and micro-heterogeneity within the 16S gene among closely related species.

Supplementary Data 7: Comparison of the 16S ONT Rapid Barcoding Kit with the in-house developed ONT RBK method and Sanger sequencing for sterile clinical samples.

| **No.** | **Sample Type** | **DNA detected following conventional 16S rDNA PCR** | **ABI 3730xl Sanger** | **ONT RBK114.96**  **(in-house ONT RBK Method)** | **ONT 16S SQK.16S114.24 -**  **(ONT 16S Barcoding all-in-one kit)** | **Total Pathogen Reads (%)** | **Clinical Notes** |
| --- | --- | --- | --- | --- | --- | --- | --- |
| 1 | Heart valve | No | N/A | *Aggregatibacter actinomycetemcomitans* | *Aggregatibacter actinomycetemcomitans* | 8% | Culture negative infective endocarditis |
| 2 | Joint Fluid - Knee | No | N/A | *Streptococcus pyogenes* | No pathogens detected | 34% | Culture negative septic arthritis |
| 3 | Joint Fluid - Knee | No | N/A | *Streptococcus pyogenes* | No pathogens detected | 10% | Culture negative septic arthritis |
| 4 | Joint Fluid - Hip | No | N/A | *Streptococcus dysgalactiae* | No pathogens detected | 33% | Culture negative septic arthritis |
| 5 | Joint Fluid - Hip | No | N/A | *Streptococcus pyogenes* | No pathogens detected | 16% | Culture negative septic arthritis |
| 6 | Heart valve | No | N/A | *Coxiella burnetii* | No pathogens detected | 1% | Culture negative infective endocarditis of a prosthetic valve - Strong positive Q Fever serology |
| 7 | Cerebrospinal Fluid | No | N/A | *Streptococcus pasteurianus* | No pathogens detected | 30% | Suspected bacterial meningitis |
| 8 | Pus - Breast abscess | No | N/A | *Corynebacterium tuberculostearicum* | No pathogens detected | 60% | Culture negative pyogenic infection |
| 9 | Pus - Pleural Fluid Empyema | No | N/A | *Fusobacterium nucleatum with mixed anaerobes and Streptococci* | *Fusobacterium vincentii* | 75% | No definitive diagnosis - Patient RIP |
| 10 | Pus - Breast abscess | No | N/A | *Corynebacterium kroppenstedtii* | N/A | 68% | Chronic granulomatous mastitis |
| 11 | Pus - Breast abscess | No | N/A | *Corynebacterium kroppenstedtii* | N/A | 44% | Chronic granulomatous mastitis |
| 12 | Pus - Breast abscess | No | N/A | *Corynebacterium kroppenstedtii* | N/A | 42% | Chronic granulomatous mastitis |
| 13 | Prevertebral tissue | Yes | No ID | Mixed skin flora including Nocardioides species | N/A | - | Large prevertebral collection, suspected MDR-TB, differential diagnosis of nocardiosis |
| 14 | Pus -Liver abscess | Yes | No ID | *Fusobacterium necrophorum* | *Fusobacterium necrophorum* | 89% | Culture negative pyogenic liver abscess |
| 15 | Pus - Pleural Fluid Empyema | Yes | No ID | *Parvimonas micra*  *Prevotella nigrescens*  *Fusobacterium species* | No pathogens detected | 66% | Culture negative empyema |
| 16 | Pus - Neck abscess | Yes | No ID | *Streptococcus pyogenes* | No pathogens detected | 83% | Culture negative pyogenic infection |
| 17 | Pus - Breast abscess | Yes | No ID | *Porphyromonas bennonis*  *Peptoniphilus grossensis*  *Finegoldia magna* | No pathogens detected | 61% | Culture negative pyogenic infection |
| 18 | Cerebrospinal Fluid | Yes | Nocardia species | *Nocardia araoensis* | No pathogens detected | 57% | Untreated HIV, disseminated nocardiosis - Culture negative |
| 19 | Pus - Breast abscess | Yes | Organisms of the Mycobacteriales order | *Corynebacterium parakroppenstedtii/ Corynebacterium kroppenstedtii* | No pathogens detected | 40% | Chronic granulomatous mastitis |
| 20 | Pus - Groin abscess | Yes | *Chlamydia trachomatis* | *Chlamydia trachomatis* | N/A | 46% | Lymphogranuloma venereum (LGV) |
| 21 | Pus - Pleural Fluid Empyema | Yes | *Clostridium perfringens* | *Clostridium perfringens* | N/A | 51% | Culture negative empyema |
| 22 | Pus - Neck Abscess | Yes | *Mixed anaerobes* | Mixed anaerobes including Anaerococcus species and Peptoniphilus species and *Finegoldia magna* | Mixed anaerobes including Anaerococcus species and Peptoniphilus species and *Finegoldia magna* | 62% | Culture negative pyogenic infection |
| 23 | Bacterial Isolate - Blood | Yes | *Clostridium subterminale* | *Clostridium culturomicium* | *Clostridium culturomicium* | 94% | Gram Positive Rods seen in anaerobic blood culture bottle |
| 24 | Pus - Pleural Fluid Empyema | Yes | *Prevotella species* | *Prevotella baroniae*  *Streptococcus anginosus* | No pathogens detected | - | Culture negative empyema |
| 25 | Cerebrospinal Fluid | Yes | *Streptococcus pneumoniae* | *Streptococcus pneumoniae* | No pathogens detected | 77% | Bacterial meningitis |
| 26 | Tissue - Ankle | Yes | *Streptococcus pyogenes* | *Streptococcus pyogenes* | *Streptococcus pneumoniae* | 86% | Orthopaedic infection |
| 27 | Pus - Elbow abscess | Yes | *Staphylococcus roterodami*  *(Staphylococcus aureus complex)* | *Staphylococcus aureus* | *Staphylococcus roterodami*  *(Staphylococcus aureus complex)* | 98% | MRSA infection |
| 28 | Aortic Tissue | Yes | *Streptococcus dysgalactiae* | *Streptococcus dysgalactiae* | *Streptococcus dysgalactiae* | 78% | Mycotic aneurysm |
| 29 | Tissue - Femur | Yes | *Staphylococcus aureus* | *Staphylococcus aureus* | No pathogens detected | 62% | Osteomyelitis |
| 30 | Joint Fluid - Knee | Yes | *Neisseria gonorrhoeae* | *Neisseria gonorrhoeae* | No pathogens detected | 68% | Culture negative septic arthritis |
| 31 | Tissue - Maxillary bone | Yes | *Streptococcus intermedius* | *Streptococcus intermedius* | *Streptococcus intermedius* | 79% | Osteomyelitis and abscesses |
| 32 | Tissue - Hip joint | Yes | *Serratia marcescens* | *Serratia marcescens* | *Serratia marcescens* | 10% | Orthopaedic infection |
| 33 | Pus - Forearm abscess | Yes | *Finegoldia species* | *Finegoldia magna* | No pathogens detected | 59% | Skin abscess |
| 34 | Heart valve | Yes | *Streptococcus sanguinis* | *Streptococcus sanguinis* | *Streptococcus sanguinis* | 70% | Culture negative infective endocarditis |

Supplementary Data 8. Total read counts of MCM2α/β reference material sequenced using MinION and Flongle flow cells with R9 chemistry. The table compares the total read counts generated from three libraries for each sequencing approach.

| MCM2α/β Reference Material | MinION Flow Cell R9 -  Total Read Count | Flongle Flow Cell R9 -  Total Read Count |  |
| --- | --- | --- | --- |
|  |  |  |  |
| Library 1 | 3.17M | 101.7k |  |
| Librart 2 | 4.54M | 127.94k |  |
| Library 3 | 1.6M | 138.46k |  |
